# Supplementary material for: Phase-Encoded Hyperpolarized Nanodiamond for Magnetic Resonance Imaging
Source: Sci Rep. 2019 Apr 11;9:5950. doi: 10.1038/s41598-019-42373-w (PMC6459867; doi:10.1038/s41598-019-42373-w)
Supplement: Supplementary file 1 — Supplementary Information [file 41598_2019_42373_MOESM1_ESM.pdf]

Supplementary Note 1: Hyperpolarization Spectra/Mechanism

Establishing the precise mechanism of ^{13}C hyperpolarization in diamond particles at $B_0 = 2.88$ T is complicated by the very similar g-factors of the electronic defects and the spin-1 ^{14}N nucleus induced hyperfine splitting of substitutional nitrogen defects. These factors mean that the ^{13}C hyperpolarization we observe at 80.87 GHz and 80.94 GHz results from unresolved, overlapping positive and negative DNP peaks from multiple EPR lines. For example, at 80.87 GHz we are observing positive hyperpolarization at $\omega_\mathrm{e} - \omega_\mathrm{n}$ from the broad component, narrow component and $m_I = 0$ line of the P1 centers plus negative hyperpolarization at $\omega_\mathrm{e} + \omega_\mathrm{n}$ resulting from the m_I = -1 line of the P1 centers. The hyperpolarization behaviour at the outer two frequencies of 80.81 GHz and 81.0 GHz is simpler, each being dominated by only one line of the P1 centers.

At 2.88 T, the ^{13}C nuclear frequency ω_n is 31 MHz, approximately twice the 0.6 mT (17 MHz) inhomogeneous linewidth Δ of P1 centres we expect at such field strengths [1]. These values are significantly larger than the homogeneous linewidth δ of the electronic defects, which for P1 centers is on the order of 600 kHz [2]. Hence, given that δ and ω_n are smaller than Δ, we expect that hyperpolarization via P1 centres, and the component of our EPR spectra with similar linewidth, occurs via the solid effect [3]. As the solid effect occurs via the interaction of one electron and one nucleus, this would also explain the roughly linear scaling of DNP enhancement with defect concentration that we observe between HPHT and NAT samples. However, we also note that the broad component of our EPR spectra is expected to have an inhomogeneous linewidth of 2.0 mT (56 MHz) at 2.88 T, larger than the nuclear Larmor frequency, raising the possibility of ^{13}C hyperpolarization via the cross effect. Other studies of diamond hyperpolarization have suggested there could also be a very small contribution to hyperpolarization from the Overhauser effect [4]. Synthesis of diamond with carefully controlled defect composition will help reveal the details of these complex DNP mechanisms.

Supplementary Note 2: Effect of T_2 relaxation times on resolution.

Even in cases of high polarization, short spin-spin coherence times limit the time available for position encoding with magnetic field gradients, limiting the achievable MRI resolution.

For conventional MRI sequences, the fundamental MRI resolution limit is reached when the NMR linewidth is approximately equal to the frequency separation between each pixel [5]:

$$\mathrm{d}z \sim \frac{1}{\gamma G \pi T_2^*} \tag{1}$$

Where $\mathrm{d}z$ is the pixel length, γ the nuclear gyromagnetic ratio, G is the peak gradient strength, and T_2^* is the transverse coherence time. Our nanodiamond samples have $T_2^* \sim 1$ ms, which corresponds to a resolution limit of 0.4 mm in our microimager (250 mT/m peak gradient strength). Increasing the resolution beyond this point will require the use of more complex MRI sequences incorporating such features as multipulse line narrowing [6].

Supplementary Note 3: ^{13}C Enrichment

Isotopic enrichment of the concentration n of spin-1/2 nuclei in nanoparticles is attractive for the proportional increase in MRI signal. Whilst increased MRI signal alone is valuable, if electron lifetimes are sufficiently short, the increase of the spin diffusion rate [7, 8] in enriched samples may also reduce the duration of DNP required to reach high nuclear polarizations. These improved properties may be achieved whilst maintaining long nuclear T_1 times, with studies of 99% ^{13}C enriched bulk diamond having shown spin-lattice relaxation times of several hours. However, in enriched samples, the dipolar coupling strength between spins increases due to decreased interspin spacing, giving an NMR linewidth $\delta_{1/2}$ that is proportional to n. Thus, increasing n carries an inversely proportional reduction in T_2^* and corresponding reductions in imaging sensitivity. In light of these considerations, we believe isotopic enrichment of spin-1/2 nuclei up to 10% abundance will reveal the ideal compromise between high magnetizations and long spin-spin relaxation times.

Supplementary Note 4: Stability of nanodiamond solutions

Dynamic light scattering (DLS) measurements showed that the 2 µm HPHT diamonds used in this work have a zeta potential of -38±7 mV in water. The 210 nm HPHT NDs have a near identical zeta potential of -39 ± 8 mV. DLS measurements of particle size and zeta potential were performed in a Zetasizer Nano ZS. Nanoparticle dispersions were prepared by sonication. DLS size measurements agreed with specifications provided by the

manufacturer. Such a large, negative zeta potential means that these HPHT diamonds are highly aggregation-resistant in aqueous solution, making them well suited to injection and biological applications that require surface functionalization [9]. The 2 μm particles display sedimentation from aqueous solution on the timescale of hours, whilst little sedimentation was observed in solutions of 210 nm HPHT NDs over a period of weeks. This difference in sedimentation phenomena occurs due to the relative size of gravitational forces and Brownian motion as we show through a calculation of the Péclet number P_e. The Péclet number is a dimensionless measure of the relative effects of flow and thermal diffusion [10] and, for a particle in suspension, is given by:

$$P_\mathrm{e} = m_\mathrm{R} g a / k_\mathrm{B} T \tag{2}$$

Where $\mathrm{m_R}$ is the buoyant mass of the particle (mass of particle minus mass of displaced solvent), g is acceleration due to gravity, a is the particle radius, k_B is the Boltzmann constant and T is the temperature. For $P_\mathrm{e} \gg 1$, gravity dominates over Brownian forces, leading to sedimentation in the absence of other hydrodynamic forces.

We can calculate a P_e of 0.003 for our 210 nm particles if we assume spherical particles, diamond density of 3510 kg m^{-3} and water density of 1000 kg/m^3. Similarly, we can calculate a P_e of 25 for the larger 2 μm diamond particles. Of interest is the critical diamond size of 504 nm where $P_\mathrm{e} = 1$, such that Brownian forces and gravitational forces are balanced. These values explain the sedimentation of the 2 μm diamond particles.

Supplementary Note 5: Conditions for adiabatic transfer

Moving hyperpolarized samples through changing magnetic fields during sample transfer from the polarizer to MRI system results in fluctuation of the Zeeman energy between spin-up and spin-down states. If the rate of change of the magnetic field is comparable to the Larmor frequency the polarization state may change. To ensure this does not occur, the adiabatic parameter A must be much less than one at all times during the transfer process. We can calculate the adiabatic parameter from [11]:

$$A = \frac{1}{\gamma_\mathrm{n} \left|\vec{B}\right|^3} \left| \vec{B} \times \frac{\mathrm{d}\vec{B}}{\mathrm{d}t} \right| \tag{3}$$

Where γ_n is the nuclear gyromagnetic ratio, $\vec{B}$ is the magnetic field and $\frac{\mathrm{d}\vec{B}}{\mathrm{d}t}$ the rate of change of the magnetic field. When $\frac{\mathrm{d}\vec{B}}{\mathrm{d}t}$ and $\vec{B}$ are parallel, as is the case when the sample enters and exits a superconducting magnet, the cross-product in Eq. 1 is zero and adiabaticity is maintained. This means that transfer is adiabatic when the sample exits the hyperpolarizer and enters the MRI system. However, we must also consider the situation where the sample enters the 380 mT Halbach array used for sample transfer, as the magnetic field in this array is perpendicular to B_0 of the superconducting NMR magnets. From an analysis of our transfer process, we estimate a minimum $\left|\vec{B}\right| = 5$ mT perpendicular to a maximum $\frac{\mathrm{d}\vec{B}}{\mathrm{d}t} = 4$ T s^{-1}. For a ^{13}C nucleus, these values give $A = 0.09$, confirming the adiabatic nature of our transfer process.

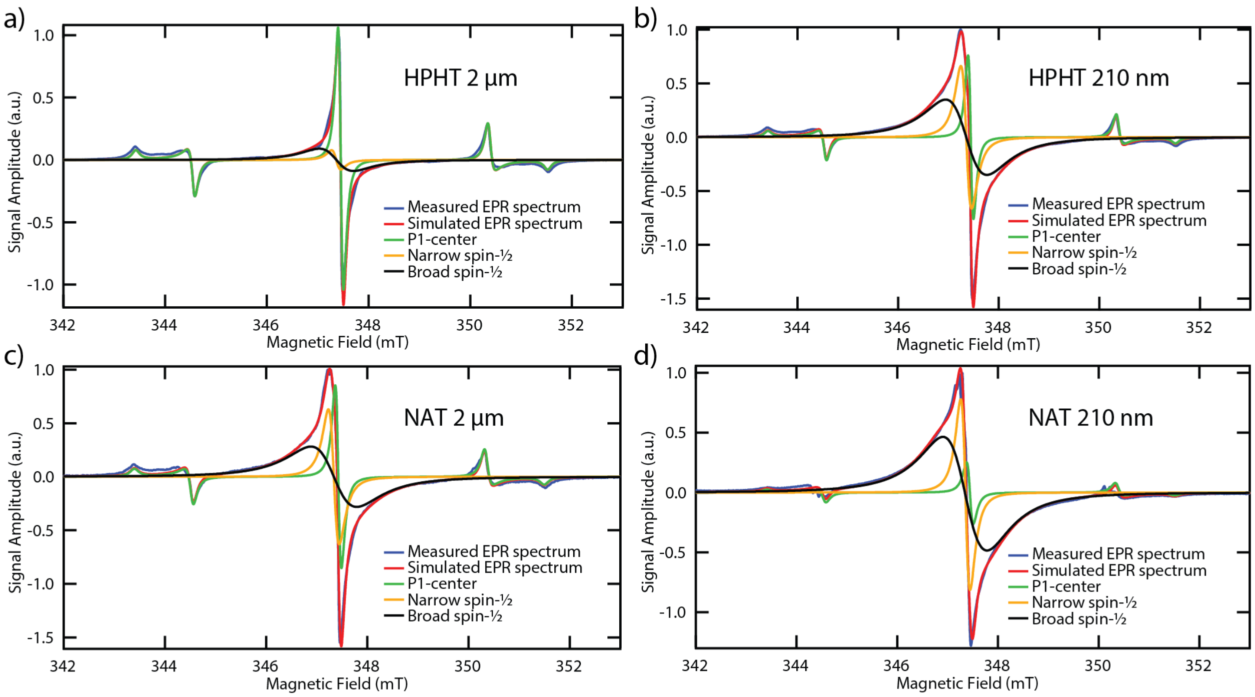

Supplementary Figure 1. **Fitting EPR spectra.** EPR spectra of HPHT 2 µm **(a)**, HPHT 210 nm **(b)**, NAT 2 µm **(c)** and NAT 210 nm **(d)** diamond particles. Spectra are fit by a spin-1/2 model comprising three components: a broad single-line, a narrow single-line and a hyperfine split P1 center. Narrow single-line (linewidth 0.2 mT) and broad single-line (linewidth approximately 0.8 mT) components likely correspond to vacancy sites and dangling bonds in the diamond lattice respectively [12, 13]. The hyperfine-split component is attributed to P1 centers (linewidth 0.1 mT), substitutional nitrogen atoms in the diamond lattice [14].

Diamond	Total (ppm)	P1 (ppm)	Broad (ppm)	Narrow (ppm)
HPHT 2 µm	210±10	95±5	108±5	7±1
HPHT 210 nm	380±20	35±2	300±20	41±2
NAT 2 µm	32±2	4.3±0.2	25±1	3.3±0.2
NAT 210 nm	190±10	6.8±0.3	169±9	14±1

Supplementary Table 1. **Defect concentrations.** Concentrations of defects extracted from the fits shown in Supp Fig. 1. The defect concentration was calculated relative to an irradiated quartz standard [15, 16].

Diamond	DNP Freq. (GHz)	a	$T_{1,\text{short}}$ (s)	$T_{1,\text{long}}(s)$
HPHT 2 μm	80.81	0.69±0.03	349 ± 28	3253±248
HPHT 2 μm	80.87	0.66±0.03	521± 46	5058±358
HPHT 2 μm	80.94	-0.63±0.02	589± 45	6330±327
HPHT 2 μm	81.0	-0.67±0.02	394±26	2966±181
NAT 2 μm	80.87	0.54±0.03	1570±145	12282±618
HPHT 210 nm	80.81	0.85±0.06	207±21	1426±332
HPHT 210 nm	80.87	0.82±0.05	278±38	3150±1188

Supplementary Table 2. **Fit parameters for hyperpolarized decays curves.** Hyperpolarized decay curves were fit with a two-component model: $M_z = ae^{-t/T_{1,\text{short}}} + (1-a)e^{-t/T_{1,\text{long}}}$, where M_z is the measured signal (proportional to the z-component of ^{13}C magnetization), $T_{1,\text{short}}$ is the relaxation time of the short component, $T_{1,\text{long}}$ is the relaxation time of the long component and a is a ratio accounting for the relative sizes of the two components. Fit parameters shown here are for hyperpolarized decay curves in Fig. 2a and Supp. Fig. 2a.

Diamond	$T_{1,\text{short}}$ (s)	$T_{1,\text{long}}(s)$	a	T_2 (ms)
HPHT 210 nm	12±5	152±40	0.42±0.09	2.1±0.1
HPHT 2 μm	77±18	6441±2220	0.59±0.05	1.2±0.1
NAT 210 nm	23±7	359±67	0.33±0.06	2.7±0.1
NAT 2 μm	491±202	9672±1607	0.29±0.06	3.1±0.1

Supplementary Table 3. **T_1 and T_2 relaxation times of thermally polarized diamond particles.** The ^{13}C T_1 relaxation times of the various diamond particles were measured using saturation recovery at $T = 293$ K and $B_0 = 7$ T. The resulting data were fit with a two component model: $M_z = 1 - ae^{-t/T_{1,\text{short}}} - (1-a)e^{-t/T_{1,\text{long}}}$, where M_z is the measured signal (proportional to the z-component of ^{13}C magnetization), $T_{1,\text{short}}$ is the relaxation time of the short component, $T_{1,\text{long}}$ is the relaxation time of the long component and a is a ratio accounting for the relative sizes of the two components. The ^{13}C T_2 relaxation times of the various diamond particles were measured at $T = 293$ K and $B_0 = 7$ T via a Hahn-echo experiment. The normalized data at various echo time were fit to an exponential model: $M_x = e^{-t/T_2}$, where T_2 is the spin-spin relaxation time.

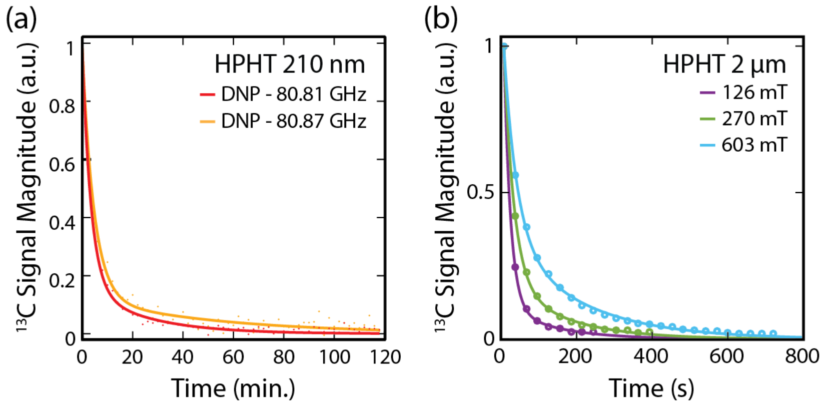

Supplementary Figure 2. **Decay of hyperpolarization.** **(a)** Signal decay of hyperpolarized 210 nm HPHT ND at $T = 293$ K and $B_0 = 7$ T. Decay curves are shown for ND hyperpolarized at 80.87 GHz (yellow) and 80.81 GHz (red). Double exponential fits to the curves are shown (see Supp. Table 2 for fit parameters). **(b)** Signal decay of hyperpolarized 2 µm HPHT ND at $T = 293$ K and 126 mT (purple), 270 mT (green) and 603 mT (blue). Decay curves are shown for ND hyperpolarized at 80.87 GHz. Double exponential fits to the curves are shown (Fit values are those shown in Fig 2b).

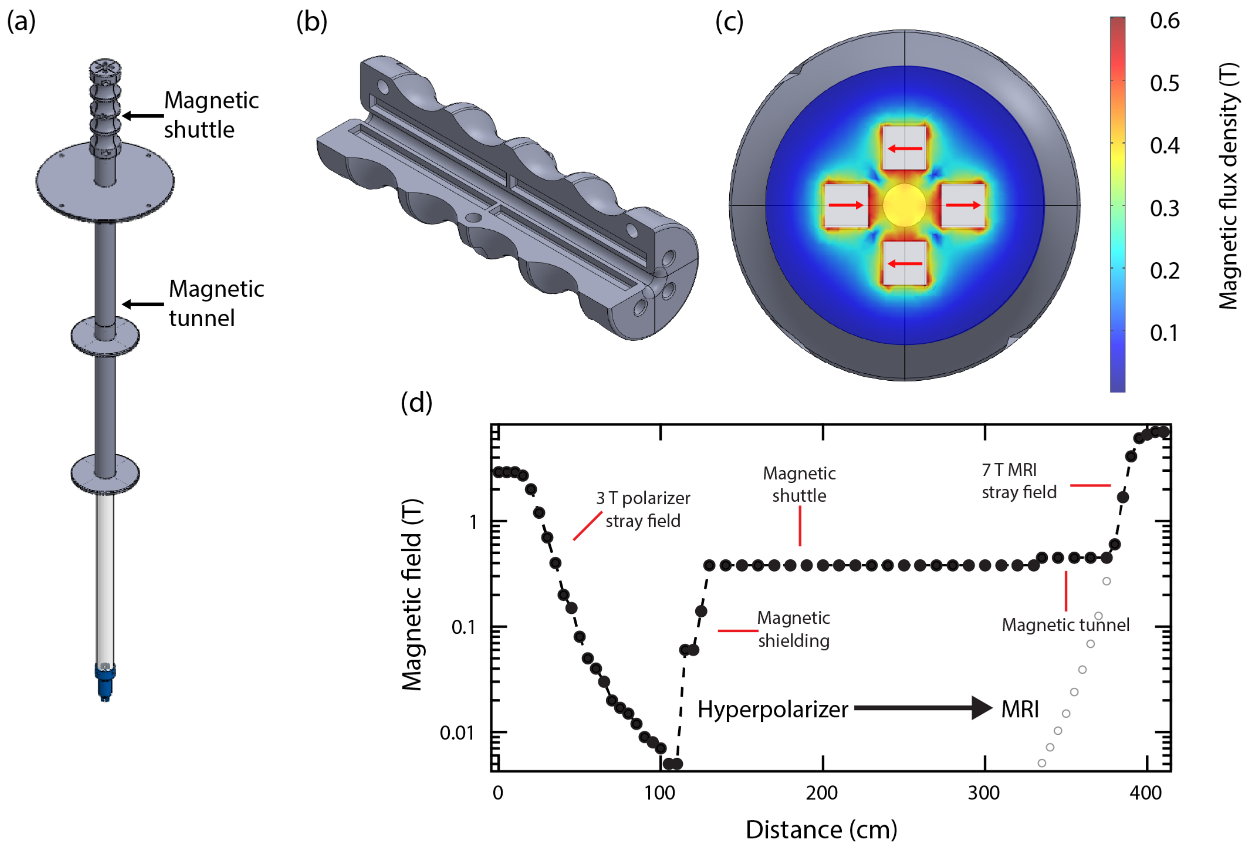

Supplementary Figure 3. **Magnetic transfer apparatus and field profile (a)** A magnetic tunnel is used to keep the sample at high magnetic field when entering the bore of the 7 T MRI. The tunnel is lined with neodymium magnets in a simplified Halbach arrangement. **(b)** Magnetic shuttle. Cutout shows slots for 5 mm x 5 mm x 60 mm neodymium magnets. **(c)** Magnetic field simulations of the magnetic field in a cross-section of the sample shuttle. Simulations were performed with Comsol Multiphysics for N38 grade neodymium magnets. Red arrows indicate orientation of magnets. **(d)** Magnetic field strength at the diamond sample during direct transfer between the hyperpolarizer and MRI. Solid circles indicate field strength with the magnetic tunnel in place. Open circles indicate the stray field from the MRI magnet when the magnetic tunnel is not in place.

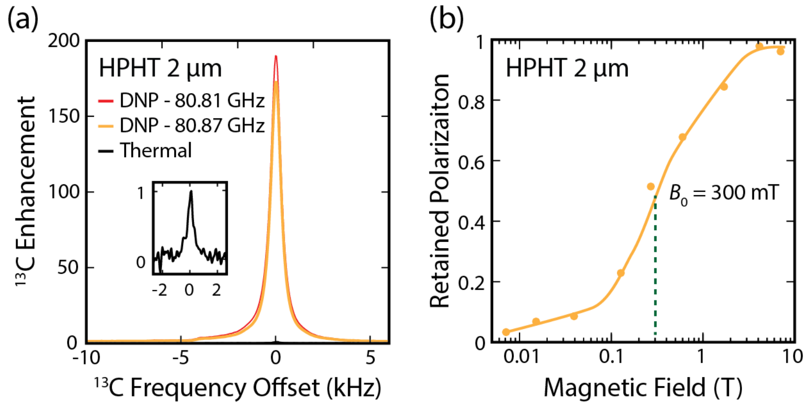

Supplementary Figure 4. **Polarization loss at low magnetic field.** **(a)** Hyperpolarized NMR spectra in the 7 T imager at room temperature after transfer of 2 μm diamond particles hyperpolarized for 20 minutes at 80.81 GHz (red) and 80.87 GHz (yellow). Magnitude is normalized to the thermal signal after 5 hours buildup at 293 K and 7 T (black). Average hyperpolarized signal magnitudes were 192±63 and 176±38 for samples hyperpolarized at 80.81 GHz and 80.87 GHz respectively. Magnitude values quoted are the average of 3 consecutive transfers with errors. The hyperpolarized signal post-transfer corresponds to ∼0.1% ^{13}C polarization. This post-transfer polarization corresponds to a near 99% reduction from the pre-transfer polarization, which we attribute to an unshielded, <5 mT, region the sample must travel through on exiting the polarizer cryostat in our homebuilt system. See the field path in Supp. Fig. 3. **(b)** ^{13}C magnetization retained after 30 seconds at various field strengths for 2 μm HPHT diamond hyperpolarized at 80.87 GHz. Solid line is intended as a guide to the eye. Data was acquired by shuttling the hyperpolarized sample between the detection coil at 7 T and the low field region. This result implies that construction of a polarizer that keeps the sample at a field strength of above 300 mT at all times would allow the hyperpolarized sample to retain 50% of its ^{13}C polarization. The construction of such polarizers is a subject of active research [11] and could see an improvement in MRI sensitivity of 50 times that seen in Fig. 3.

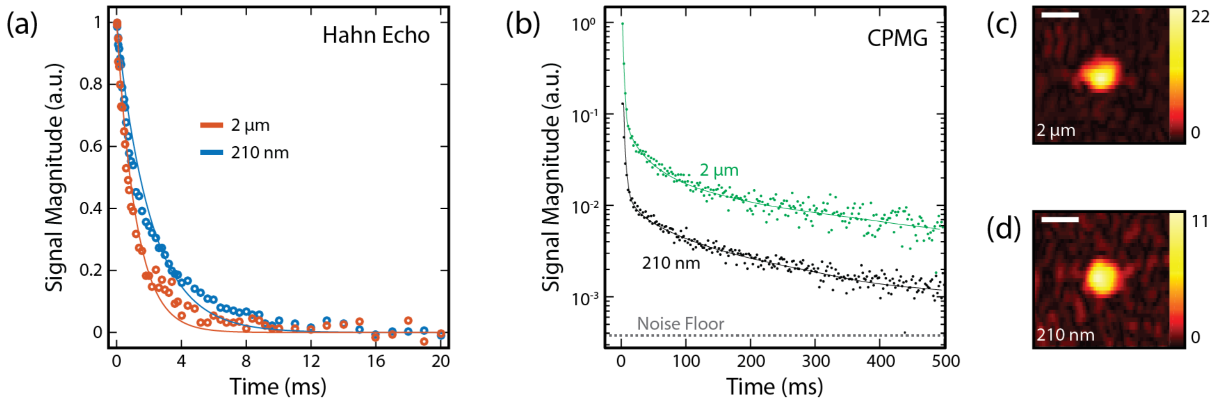

Supplementary Figure 5. **Spin-echoes in diamond.** **(a)** ^{13}C T_2 characterization of HPHT diamonds in a Hahn-echo experiment at $B = 7$ T and $T = 293$ K. Data is shown for 2 µm (red) and 210 nm (blue) samples. Solid lines are a fit to the experimental data with fit parameters shown in Supp. Table 3. The 2 µm fit is shown in Fig. 3. **(b)** Signal magnitude of hyperpolarized echoes in a CPMG experiment with 2.1 ms echo spacing. Traces for hyperpolarized 2 µm (green) and 210 nm (black) HPHT samples are shown. **(c)** Rapid Acquisition with Refocused Echoes (RARE) acquired image of hyperpolarized HPHT 2 µm diamonds at 200 mg/mL. **(d)** RARE acquired image of hyperpolarized HPHT 210 nm diamonds at 200 mg/mL. All RARE data were acquired with a 64 $\times$ 32 matrix and pixel resolution of 0.7 mm $\times$ 0.5 mm resolution. A short pulse width of 40 µs and 2.1 ms echo time was used to allow gradient ramping with resolution similar to that used in GRE acquisitions in Fig. 3. Complete RARE timing parameters are provided in Supp Fig. 6. To prevent artifacts due to rapid initial signal decay, centrically ordered phase encoding begins with the third CPMG echo and post-processing was used to weight the amplitude of phase encodes by the gradient-free long tails acquired in **b**. Scale bars are 3 mm in length. The colorscale in all images indicates signal magnitude in units of the noise floor. The SNR of the RARE images shown here should be multiplied by 2.5$\times$ for a fair sensitivity comparison with GRE images shown in Fig. 3 (to take account of variations in pixel size and the expected SNR with a variable flip angle GRE sequence). Hence, the sensitivity of GRE and RARE sequences are nearly equivalent as we have applied them in our MRI scanner. However, with higher strength gradients it would be possible to shorten the 2.1 ms echo time and leverage the significantly larger ^{13}C signal tail that appears at 0.5 ms echo time, as shown in Fig. 2c.

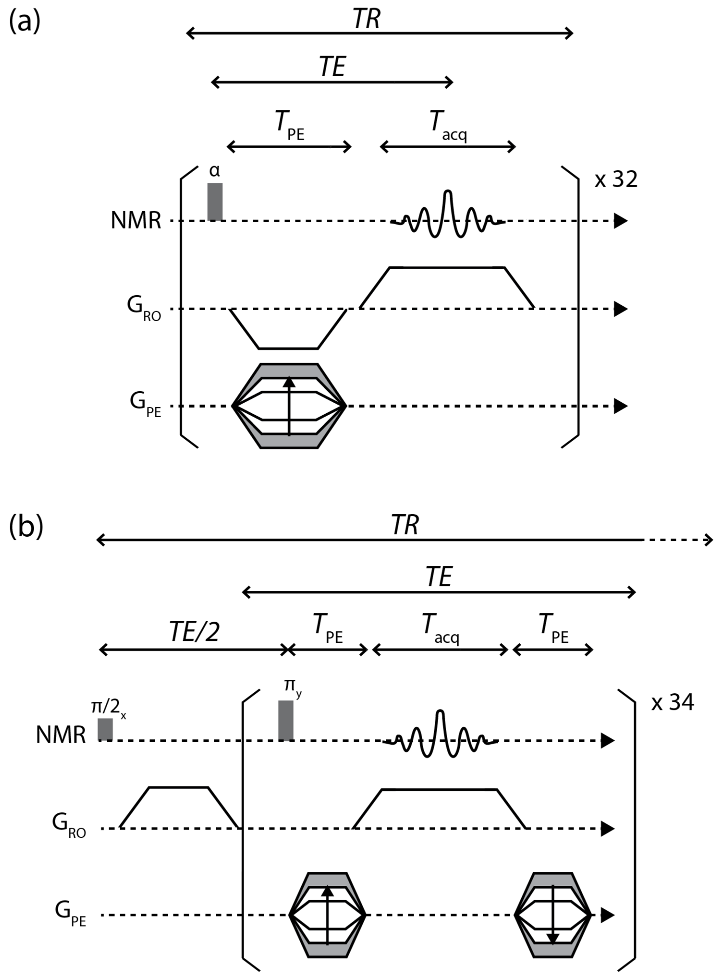

Supplementary Figure 6. **Imaging Sequences.** **(a)** Gradient Refocused Echo (GRE) sequence used for ^{13}C imaging. Relative timing of NMR pulses, readout gradient (G_{RO}) and phase encode gradient (G_{PE}) are shown. Imaging parameters were: repetition time (TR) = 100 ms, echo time (TE) = 1.2 ms, acquisition time (T_{acq}) = 0.64 ms, phase encode time (T_{PE}) = 0.4 ms and $\alpha = 20°$. No crusher gradients were required as the 100 ms repetition time is sufficient to spin dephasing with negligible spin-lattice relaxation. TE and T_{PE} were adjusted to 0.9 ms and 0.12 ms respectively for mouse experiments. **(b)** Rapid acquisition with refocused echoes (RARE) sequence used for ^{13}C imaging. Relative timing of NMR pulses, readout gradient (G_{RO}) and phase encode gradient (G_{PE}) are shown. Imaging parameters were: repetition time (TR) = 75 ms, echo time (TE) = 2.1 ms, acquisition time (T_{acq}) = 0.8 ms and phase encode time (T_{PE}) = 0.4 ms. Gradient amplitude was set to zero for the first 2 signal acquisition steps so that imaging began after the long tail echo was established.

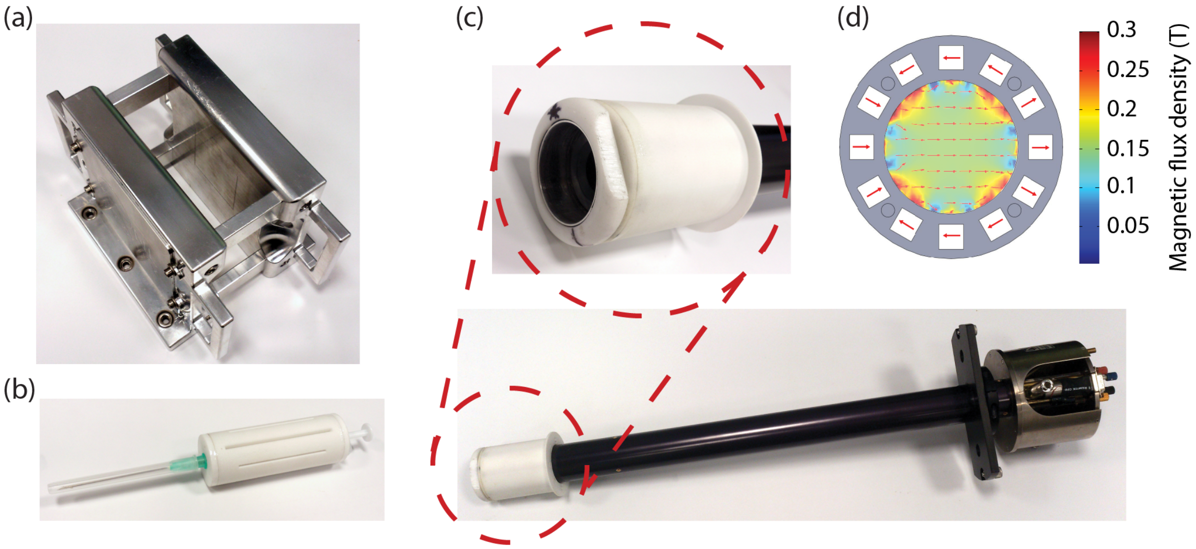

Supplementary Figure 7. **Transfer hardware for animal imaging.** Various magnet assemblies used to maximize the magnetic field at hyperpolarized samples. **(a)** Two 100 mm × 100 mm neodynium magnets create a 50 mm × 50 mm × 50 mm working space with a magnetic field greater than 200 mT. **(b)** Syringe with simplified Halbach array for injection of hyperpolarized diamonds. **(c)** Microimaging probe with a sliding collar that integrates a Halbach array. **(d)** Simulations (Comsol Multiphysics) of the magnetic field strength inside simplified Halbach array with an inner diameter of 30 mm.

Supplementary References

---

[1] Yavkin, B. V., Mamin, G. V., Gafurov, M. R. & Orlinskii, S. B. Size-dependent concentration of N^0 paramagnetic centres in HPHT nanodiamonds. *Magn. Reson. Solids* **17**, 15101 (2015).

[2] Van Wyk, J. A., Reynhardt, E. C., High, G. L. & Kiflawi, I. The dependences of ESR line widths and spin - spin relaxation times of single nitrogen defects on the concentration of nitrogen defects in diamond. *J. Phys. D. Appl. Phys.* **30**, 1790–1793 (1997).

[3] Maly, T. *et al.* Dynamic nuclear polarization at high magnetic fields. *J. Chem. Phys.* **128**, 052211 (2008).

[4] Bretschneider, C. O. *et al.* On The Potential of Dynamic Nuclear Polarization Enhanced Diamonds in Solid-State and Dissolution ^{13}C NMR Spectroscopy. *ChemPhysChem* **17**, 2691–2701 (2016).

[5] Strange, J. H. & Halse, M. R. Imaging Techniques for Solids and Quasi-Solids. *eMagRes* 1–9 (2007).

[6] Frey, M. a. *et al.* Phosphorus-31 MRI of hard and soft solids using quadratic echo line-narrowing. *Proc. Natl. Acad. Sci. U.S.A.* **109**, 5190–5195 (2012).

[7] Shabanova, E., Schaumburg, K. & Sellschop, J. P. F. ^{13}C NMR Investigations of SpinLattice Relaxation in 99% ^{13}C-Enriched Diamonds. *J. Magn. Reson.* **130**, 8–17 (1998).

[8] Terblanche, C. J., Reynhardt, E. C., Rakitianski, S. A. & Van Wyk, J. A. ^{13}C Spin-Lattice Relaxation in Natural Diamond: Zeeman Relaxation in Fields of 500 to 5000 G at 300 K Due to Fixed Paramagnetic Nitrogen Defects. *Solid State Nucl Magn Reson.* **19**, 107–129 (2001).

[9] Mochalin, V. N., Shenderova, O., Ho, D. & Gogotsi, Y. The properties and applications of nanodiamonds. *Nat. Nanotechnol.* **7**, 11–23 (2012).

[10] Ramaswamy, S. Issues in the statistical mechanics of steady sedimentation. *Adv. Phys.* **50**, 297–341 (2001).

[11] Milani, J. *et al.* A magnetic tunnel to shelter hyperpolarized fluids. *Rev. Sci. Instrum.* **86**, 024101 (2015).

[12] Loubser, J. H. N. & Wyk, J. A. Electron spin resonance in the study of diamond. *Rep. Prog. Phys.* **41**, 1201–1248 (1978).

[13] Zaitsev, A. *Optical Properties of Diamond, A Data Handbook* (Springer, 2001).

[14] Barklie, R. C. & Guven, J. ^{13}C hyperfine structure and relaxation times of the P1 centre in diamond. *J. Phys. C Solid State* **14**, 3621–3631 (1981).

[15] Eaton, G. R. *et al.* A signal-to-noise standard for pulsed EPR. *J. Magn. Reson.* **205**, 109–113 (2010).

[16] Eaton, G. R., Eaton, S. S., Barr, D. P. & Weber, R. T. *Quantitative EPR* (Springer, 2010).
